# Microtubule Affinity-Regulating Kinase 4 Promotes Oxidative Stress and Mitochondrial Dysfunction by Activating NF-κB and Inhibiting AMPK Pathways in Porcine Placental Trophoblasts

**DOI:** 10.3390/biomedicines10010165

**Published:** 2022-01-13

**Authors:** Liang Tian, Guangfan Liu, Ziqi Kang, Peishi Yan

**Affiliations:** Department of Intelligent Animal Husbandry and Environmental Science, College of Animal Science and Technology, Nanjing Agricultural University, Nanjing 210095, China; 2021105063@stu.njau.edu.cn (G.L.); 2021105027@stu.njau.edu.cn (Z.K.); yanps@njau.edu.cn (P.Y.)

**Keywords:** MARK4, oxidative stress, mitochondria, NF-κB, placenta, AMPK, pig

## Abstract

The aim of this investigation was to evaluate the role of MARK4 in the regulation of oxidative stress and mitochondrial dysfunction in pig placental trophoblasts and analyze the signaling pathways involved. In this study, we found that enhanced MARK4 contributed to augmented oxidative stress in pig trophoblasts, as evidenced by decreased total antioxidant capacity (TAC); higher production of reactive oxygen species (ROS); elevated protein carbonylation; and reduced SOD, CAT, and GSH-PX activities. Further analyses revealed MARK4 impaired mitochondrial oxidative respiration in cultured trophoblasts, which was associated with reduced ATP content, decreased mitochondrial membrane potential, lower mitochondrial Complexes I and III activities, and down-regulated protein contents of subunits of complexes I, II, and V. At same time, mitochondrial biogenesis and structure were negatively altered by elevated MARK4. By antioxidant treatment with vitamin E (VE), oxidative stress along with impaired mitochondrial function induced by enhanced MARK4 were blocked. Furthermore, we found activation of AMPK signaling prevented MARK4 from blocking mitochondrial biogenesis and function in pig trophoblast cells. Finally, we demonstrated that the IKKα/NF-κB signal pathway was involved in MARK4 activated oxidative stress and mitochondrial dysfunction. Thus, these data suggest that MARK4 promotes oxidative stress and mitochondrial injury in porcine placental trophoblasts and can contribute to the developing of knowledge of pathological processes leading to mitochondrial dysfunction associated with excessive back-fat in the pig placenta and to the obesity-associated pregnant syndrome.

## 1. Introduction

Maternal obesity has been demonstrated to induce a lipotoxic milieu within the placenta characterized by increased placental lipid, inflammation, and oxidative stress [1,2], along with increased concentrations of inflammatory cytokines and fatty acids in maternal plasma [2,3], resulting in placental dysfunction and therefore the adverse perinatal outcomes in human beings and some animal species like the pig [1,4]. Lipotoxicity has been shown to provoke cellular dysfunction through increased production of reactive oxygen species (ROS) and the activation of inflammation in highly metabolically active tissues such as adipose, skeletal muscle, and placenta [2,5,6]. Much of the evidence supporting an adverse effect of exaggerated oxidative stress in obese pregnancy is based on placental oxidative damage that induce placental dysfunction [7,8], and recent evidence further revealed that increased ROS generation and mitochondrial abnormalities are major underlying mechanisms of the physiopathology of pregnancies complicated by maternal obesity or diabetes [9,10]. Indeed, as a key energy source for placental function, mitochondria is highly susceptible to oxidative stress damage, resulting in adverse changes that affect mitochondrial structure and function [11]. Several studies have reported that maternal obesity contributes to decreased mitochondrial content and lower mitochondrial respiration and higher generation of superoxide in the human placenta, thus leading to compromised fetal growth and development [12]. Furthermore, our previous data indicated that excessive back-fat leads to increased ROS production, impaired mitochondrial respiration, and mitochondrial abnormalities in content and structure in term placenta from sows, suggesting that maternal obesity could induce mitochondrial dysfunction in the full-term porcine placenta [13]. However, the exact cellular and molecular mechanisms responsible for excessive back-fat associated mitochondrial injury in the pig placenta remain elusive.

Microtubule affinity-regulating kinase 4 (MARK4), a member of the microtubule affinity-regulating kinases (MARKs) family, has been demonstrated to participate in diverse physiological processes, including fertility [14], immune system homeostasis [15], and regulation of programmed cell death [16]. As the mammalian homologs of nematode Par-1, MARK4 shares similar structure with other family members (MARK1(Par-1c), MARK2(Par-1b/EMK1), and MARK3(Par-1a/C-TAK1), which consist of three distinct domains, namely, a catalytic kinase domain, a ubiquitin-associated domain, and a kinase associated domain [17]. Several studies have shown that MARK4 knockout mice is protected from obesity and insulin resistance (IR) induced by high-fat diet (HFD), suggesting a novel role of MARK4 in modulating glucose homeostasis and energy metabolism [18]. Recently, Mark4 has been shown to promote adipogenesis and trigger adipocytes apoptosis, by activating JNK1 and inhibiting p38MAPK pathways, and induce oxidative stress and inflammation by binding to PPARγ and activating the NF-κB pathway in mice adipocytes [19,20]. In addition, our previous findings revealed that excessive back-fat is coupled with increased activation of MARK4 in pig term placenta [21], and our in vitro data further demonstrated that MARK4 is involved in regulating lipogenesis of pig placental trophoblasts via activating WNT/β-catenin signal pathway, suggesting MARK4 has potential as a causal mechanism to impact placental lipotoxicity associated excessive back-fat [22]. Thus, MARK4 can be a versatile protein implicated in large number of metabolic or physiopathologic processes and could be considered as a potential regulator of placental dysfunction associated with excessive back-fat in the pig placenta. However, the regulatory role of MARK4 on mitochondrial dysfunction in porcine placenta associated with excessive back-fat is still unknown, as warrants further studies.

In this study, using an in vitro cell model (pig placental trophoblasts), we found that MARK4 accentuated the oxidative stress upon the status of lipotoxic insult and induced impaired mitochondrial respiration and negative alterations in mitochondrial biogenesis and structure. In addition, we demonstrated that, through activating the IKKα/NF-κB and inhibiting the AMPK signal pathways, MARK4 promoted oxidative stress and mitochondrial dysfunction in pig placental trophoblasts. These findings illustrate MARK4 may serve as a potential regulator of mitochondrial injury associated with excessive back-fat in the pig placenta and contribute to a better understanding of the background of pathological processes leading to obesity-associated pregnant syndrome.

## 2. Materials and Methods

### 2.1. Reagents and Cell Culture

Before performing the isolation and culture of porcine placental trophobalst cells (cytotrophoblasts), the following reagents need to be purchased, including Ham’s F12/Dulbecco’s Modified Eagle Medium (DMEM/F12) (HyClone, Logan, UT, USA), fetal bovine serum (FBS) (HyClone, Logan, UT, USA), Trypsin (Gibco, Grand Island, NY, USA), DNase I (Roche, Basel, Switzerland), Phosphate-buffered saline (PBS) (Life Technologies, Grand Island, NY, USA), Bovine serum albumin (BSA) (Amresco, Solon, OH, USA); Percoll (Pharmacia, London, UK), 10 × Hanks balanced salt solution (HBSS) (Gibco, Grand Island, NY, USA), 100 × Penicillin-Streptomycin (10,000 U/mL) (Invitrogen, Carlsbad, CA, USA), 100 × Insulin–Transferrin–Selenium (ITS; Sigma, Saint Louis, MO, USA) and epidermal growth factor (EGF; Invitrogen, Carlsbad, CA, USA).

Placental villous tissues of *Landrace* sows were harvested from vaginal delivery; visible fibers and fetal amnion were removed, and the villous tissue was washed thoroughly in cold PBS containing 100 U/mL penicillin and 100 μg/mL streptomycin. Then the placental villous tissue was minced into 1–3 mm^3^ pieces with scissors and incubated in 20 mL digestion medium composed of DMEM/F12 containing 0.125% trypsin, 20 U/mL DNase I, and 0.1% BSA for 30 min at 37 °C with continuous shaking. After the incubation, 37 °C preheated FBS was added to the digestion flask to stop trypsinization. Flask contents were mixed and filtered through a 70 μm cell strainer to remove undigested tissues and large cell aggregates. The filtered cells were further purified by a discontinuous 35% and 45% (*v/v*) Percoll gradient centrifugation at 2000× *g* for 20 min. Villous cytotrophoblasts were then collected from the appropriate layers between 35% and 45% Percoll density gradient separated layers and cultured in DMEM/F12 supplemented with 10% FBS, 1% (*v/v*) ITS, 10 ng/mL of EGF, 100 U/mL penicillin, and 100 μg/mL streptomycin at 37 °C under 5% CO_2_ as previously described [13]. The purity of cytotrophoblasts isolated from full-term placentas was determined by flow cytometry as previously described [23], using FITC fluorescein-labeled antibody against cytokeratin-7 (Santa Cruz Tech, Dallas, CA, USA) as a specific marker of trophoblast cells.

### 2.2. Transfection of Cytotrophoblasts with Plasmids

Transfection-ready DNA constructs of Myc-MARK4, mtEGFP, or mtDsRed2 (mitochondrial green or red fluorescent protein) were made by Generay Biotech Company (Shanghai, China) using pcDNA3.1 expression vector. shRNA sequences against MARK4 were contrived and synthesized by Genepharma Company (Shanghai, China) using pGpU6/Neo shRNA expression vector named sh1-MARK4, sh2-MARK4, and sh3-MARK4. After transfection efficiency detection (Figure S1), the optimal shRNA of MARK4 was chosen and named sh-MARK4. pcDNA3.1-vector and negative-shRNA were used as control vectors. The X-treme GENE HP Reagent (Roche, Basel, Switzerland) was used in cell transfection with plasmids. Cytotrophoblasts were plated at a concentration of 6 × 10^5^–2 × 10^6^/dish in 60-mm dishes. 2 µg DNA was mixed with Opti-MEMI media (Invitrogen, Carlsbad, CA, USA) and X-treme GENE HP Reagent and then added into each dish for 24 h or 48 h to allow the expression of DNA or shRNA constructs according to the protocol.

### 2.3. Cell Viability Assay and Drug Treatment

**Figure 1:**
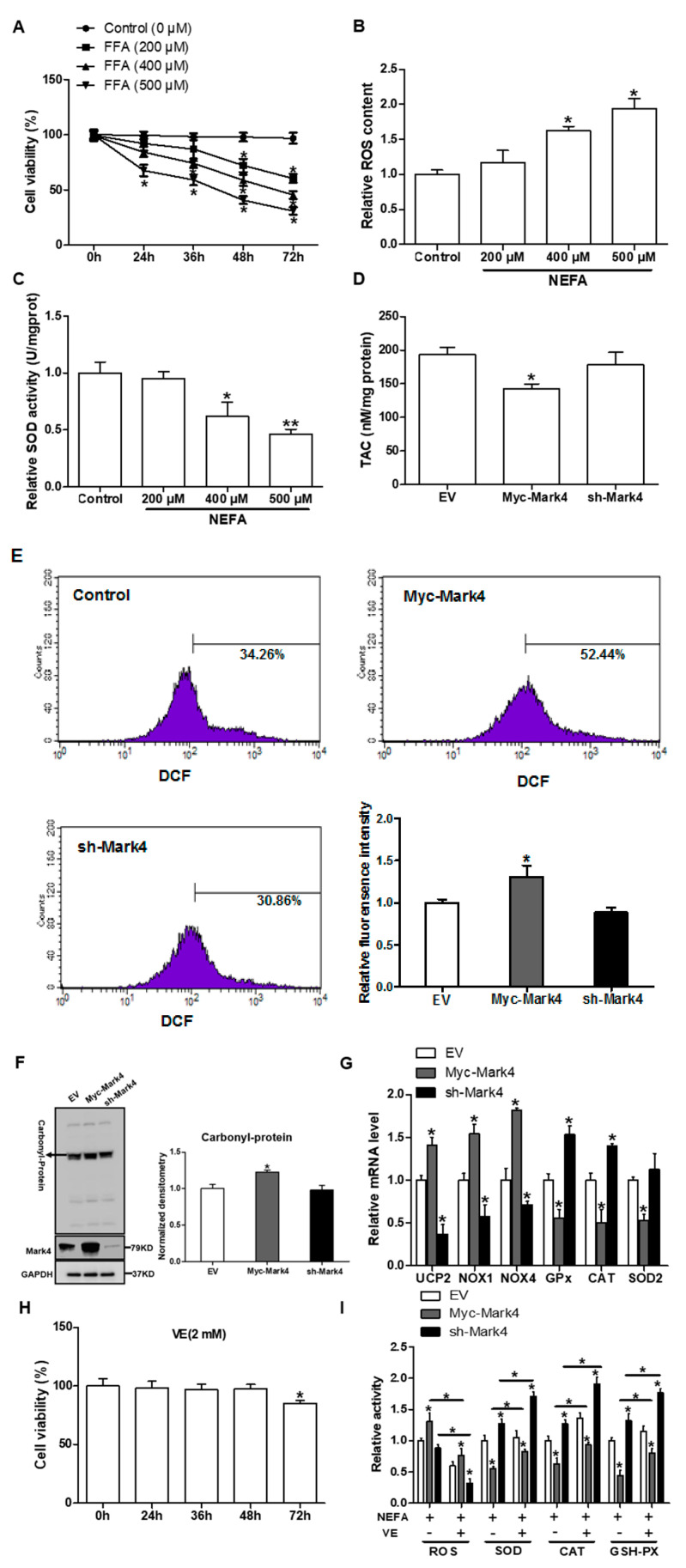
MARK4 promotes oxidative stress in pig primary trophoblast cells. (**A**) Primary cytotrophoblasts isolated from pig placentas were cultured and incubated for 0, 24, 36, 48, and 72 h in the presence of 200, 400, or 500 μM NEFA. Cell viability was detected by CKK-8 (*n* = 3). (**B**,**C**) The relative ROS (B) and SOD (C) activity of the primary cytotrophoblasts incubated for 24 h in the presence of 200, 400 or 500 μM NEFA (*n* = 3). (**D**) Total antioxidant capacity (TAC) was measured in primary trophoblast cells after transfection with Myc-MARK4 and sh-MARK4 for 48 h. Cells were then incubated with 400 μM NEFA for 24 h (*n* = 3). (**E**) ROS content, evaluated by DCF production using flow cytometry or a spectrophotometer (*n* = 3), respectively, in pig cytotrophoblasts after transfection with Myc-MARK4 and sh-MARK4 for 48 h. Before assessment of ROS generation, cells were incubated with 400μM NEFA for 24 h. Value in scale bar depicts the percentage of DCF-positive cells. (**F**) Representative immunoblots and densitometric quantification for total protein carbonylation (*n* = 3). (**G**) Relative mRNA expression of oxidant stress-related genes after transfection with Myc-MARK4 and sh-MARK4 for 48 h in cytotrophoblasts. Cells were then treated with 400 μM NEFA for 24 h (*n* = 3). (**H**) Isolated primary trophoblast cells were cultured and incubated for 0, 24, 36, 48, and 72 h in the presence of 2 mM VE. Cell viability was detected by CKK-8 (*n* = 3). (**I**) Relative activity of ROS, SOD, CAT, and GSH-PX after transfection with Myc-MARK4 and sh-MARK4 for 48 h in trophoblast cells. Before the assessment, cells were incubated with 400 μM NEFA or 2 mM VE for 24 h (*n* = 3). Results are expressed as mean ± SEM. ** *p* < 0.01; * *p* < 0.05 compared with the control group. ROS: reactive oxygen species; DCF: dichlorofluorescein; SOD: superoxide dismutase; GSH-PX: glutathione peroxidase; CAT: catalase; NEFA: non-esterified fatty acid; Myc-MARK4 group: over expression of MARK4 group; sh-MARK4 group: knock down of MARK4 group; Control: empty vector (EV) group.

Cell viability assay was performed by CCK-8 cell counting kit (KeyGen BioTECH, Nanjing, China) according to the manufacturer’s instructions as previously described [22]. Briefly, the treated cells were seeded in 96-well plate at a density of 1 × 10^4^ and cultured with 0, 200, 400, or 500 µM fatty acid (FA) and 2 mM Vitamin E (VE) for the amount of time specified in Figure 1, respectively. A volume of 10 µL CCK-8 solution was added into each well and incubated for 2 h at 37 °C. Absorbance assay was performed at 450 nm using a Multiskan Go Microplate Spectrophotometer (Thermo Scientific, Waltham, MA, USA).

In order to induce mitochondrial oxidative injury in cytotrophoblasts in vitro, cells were treated with 400 µM Fatty Acid Supplement (2 mol of linoleic acid and 2 mol of oleic acid per mole of albumin) (L9655; Sigma-Aldrich, St. Louis, MO, USA) in triplicate as previously described [13]. Treatment media without fatty acids was added with bovine serum albumin (FA free) to maintain the same osmolarity. Furthermore, in order to reveal the molecular mechanism of signal pathways, three groups of cells in cell experiments in vitro were treated with one of the following specific agonists or inhibitors: 1 mM AICAR (AMPK agonist; MCE, Shanghai, China) or 50 μM DHMEQ (NF-κB-specific inhibitor; MCE, Shanghai, China) for the amount of time specified in the individual figures.

### 2.4. Measurement of Oxidative Stress

The intracellular level of ROS production was tested using a cell-permeable non fluorescent probe 2′, 7′-dichlorofluorescin diacetate (DCFH-DA) (KeyGen BioTECH, Nanjing, China) as previously described [13]. Briefly, cytotrophoblasts were treated with FA (0, 200, 400, and 500 µM) or drug treatment for 24 h. The treated cells were then harvested, washed with cold PBS, and incubated with 10 µM DCFH-DA 37 °C for 30 min. The production of ROS was examined using a Luminescence Spectrophotometer (Promega Corporation, Madison, WI, USA) or flow cytometry by measuring the fluorescence intensity of dichlorofluorescein (DCF) at an excitation wavelength of 488 nm and emission wavelength of 530 nm or DCF fluorescence-activated cell sorting according to the manufacturer’s protocol.

For the key oxidative enzyme activity detection, cells were harvested after the culture medium removed, washed with ice-cold PBS three times and lysed with cell lysis buffer (20 mM Tris, 150 mM NaCl, 1% Triton X-100). The lysate was centrifuged at 10,000× *g* for 5 min at 4 °C. Then superoxide dismutase (SOD) activity, total antioxidant capacity (TAC), catalase (CAT) activity, and glutathione peroxidase (GSH-PX) activity assays were performed using the commercially available kits (Jiangcheng Bioengineering Institute, Nanjing, China) as previously described [24].

### 2.5. Mitochondrial Analysis

Fluorescent probe MitoTracker Red (KeyGen BioTECH, Nanjing, China) was used to estimate mitochondrial biogenesis as previously described [25]. Cells were harvested, washed with cold PBS, and incubated at 37 °C for 20 min with 100 nM MitoTracker Red. Meanwhile, the cell nuclei were counterstained with 40, 6-diamidino-2-phenylindole (DAPI) for 10 min. After incubation, the cells were mounted on glass slides and examined on confocal laser scanning microscope (Zeiss LSM 700 META, Jena, Germany). Quantification of the fluorescence intensity from the red channel (MitoTracker Red) was performed using the Image J software (NIH Image). For mitochondrial DNA (mtDNA) copy number analysis, total DNA was extracted from treated cells in the individual figures (*n* = 3 from each group), using TIANamp Genomic DNA Kit (TIANGEN BIOTECH, Beijing, China). A pair of primers (Table S1) corresponding to ND1 and Cyclophilin-A were used to amplify a mitochondrial and nuclear DNA fragment, respectively. The relative mtDNA copy number was analyzed by 2^−ΔΔCt^ method as previously described [21]. For Citrate synthase (CS) activity measurement, cytotrophoblasts were solubilized in ice-cold extraction buffer, and the cell lysate was centrifuged at 16,000× *g* for 20 min at 4 °C. Then CS activity was measured in the supernatant using a commercial kit from Sigma (CS0720; Shanghai, China) by a spectrophotometer method as previously reported [13].

For mitochondrial respiration assessment, cytotrophoblasts were suspended in 2 mL DMEM/F12 culture medium and measured by a Seahorse XF24 analyzer (Seahorse Biosciences, Shanghai, China) as previously described [13]. Oxygen consumption rates (OCR) was normalized to total cellular protein. Protein concentration was detected using Pierce BCA Protein Assay Kit (Thermo Scientific, Waltham, MA, USA). For mitochondrial membrane potential (ΔΨm) assay, cells were harvested, washed with cold PBS, and incubated at 37 °C for 15 min with 5 µg/mL fluorescent probe JC-1 (KeyGen BioTECH, Nanjing, China) as previously described [20]. Cells were then analyzed by fluorescence-activated cell sorting using flow cytometry. The stained cells with high mitochondrial membrane potential (red fluorescence) were present in the upper right (UR) quadrant of the FACS histogram.

Mitochondrial complexes activity and ATP production rate were measured on isolated mitochondria from cell homogenate using Mitochondria Isolation Kit (KeyGen BioTECH, Nanjing, China) as previously described [21]. Briefly, cytotrophoblasts were homogenized in ice-cold isolation buffer, then centrifuged at 1200× *g* for 5 min at 4 °C. The supernatant was collected and centrifuged at 7000× *g* for 10 min at 4 °C. The resulting pellet was re-suspended in the ice-cold suspension buffer and centrifuged at 9500× *g* for 5 min at 4 °C. The mitochondria was collected in the sediments. The activities of mitochondrial complexes were determined using the Mito Complexes I and III Activity Assay Kits (GenMed Scientifics Inc., Shanghai, China) as previously reported [20]. The mitochondrial ATP production rate was detected using ENLITEN ATP Assay System (Promega; Madison, WI, USA) by 1420 Multilabel Counter (PerkinElmer; Fremont, CA, USA) according to the manufacturer’s instruction as previously described [13].

The mitochondrial fusion experiment was conducted by procedures as previously described [26]. Briefly, cells carrying different transfectants expressing mtEGFP or mtDsRed2 were washed with cold PBS, trypsinized, then mixed and incubated for 60 s with 1 mL of a preheated (37 °C) solution of PEG400 (50% [*v*/*v*] in DMEM/F12) containing 20 μg/mL cycloheximide. Cells were then washed with no serum DMEM/F12 containing cycloheximide (20 μg/mL) and recovered in the same solution for 30 min at 37 °C. After incubation, cells were washed extensively with DMEM/F12 containing 10% serum, transferred to prewarmed culture medium with cycloheximide (20 μg/mL), and plated on glass coverslips 4 h before microscopy on confocal laser scanning microscope.

### 2.6. Transmission Electron Microscopy

For the observation of alterations in mitochondrial structure and density, transmission electron microscopy was performed as previously described [13]. Briefly, after fixation procedures by glutaraldehyde (30 mg/L) and Osmium tetroxide (1%), cytotrophoblast cultures were dehydrated, and embedded in Epon-812. Cell pellet was then cut using an RMC/MTX ultramicrotome (Boeckeler, Tucson, AZ, USA). Ultrathin sections (50 nm) were mounted on copper grids, contrasted with 8% uranyl acetate and lead citrate, and observed with a JEM-1400 Plus transmission electron microscopy (Jeol LTD, Tokyo, Japan). Image analysis was performed using the Image-ProPlus 6.0 software from Media Cybernetics (Rockville, MD, USA).

### 2.7. Real-Time Quantitative PCR Analysis

Total RNA was purified from cultured cytotrophoblasts (*n* = 3 from each group) with the High Pure RNA tissue kit (Omega Bio-Tek, Norcross, GA, USA) and quantified using NanoDrop Spectrophotometer ND-1000 (NanoDrop, Wilmington, DE, USA). Two micrograms of cDNA was synthesized with PrimeScript RT Master Mix Kit (TaKaRa, Tokyo, Japan). Real-time RT-PCR was performed in 25 µL reaction system containing specific primers (Table S1) and SYBR Premix Ex Taq II (TaKaRa, Tokyo, Japan), and conducted on the Step One Plus Real-Time PCR System (ABI, Waltham, MA, USA) with the following program: 95 °C for 30 s, 95 °C for 5 s, 60 °C for 30 s, 95 °C for 15 s, 60 °C for 1 min, and 95 °C for 15 s, with 40 cycles of steps 2 and 3. Primers were synthesized by Invitrogen (Shanghai, China). The levels of mRNA were normalized in relevance to GAPDH and HPRT1. Relative gene expression was calculated using the comparative Ct method with the formula 2^−^^ΔΔCt^ [27]. The geometric mean of relative gene expression was calculated and used for further analysis as previously reported [28].

### 2.8. Protein Extraction and Western Blotting Analysis

Total protein from cultured cells (*n* = 3 from each group) was extracted using cell lysis buffer (Beyotime Biotechnology, Nanjing, China) by procedures as previously described [23]. Mitochondrial protein isolation was performed using Mitochondrial Protein Extraction Kit (KeyGen BioTECH, Nanjing, China) and suspended in lysis buffer supplemented with a protease and phosphatase inhibitor cocktail (Sigma, Saint Louis, MO, USA). The concentration of protein was quantified using BCA Protein Assay kit (Thermo Scientific, Waltham, MA, USA). For protein carbonyl derivatives detection, a standard immunoblot was developed using OxiSelect Protein Carbonyl Immunoblot Kit (Cell Biolabs, San Diego, CA, USA) following the manufacturer’s instructions as previously reported [13]. Protein samples (50 µg) were separated by electrophoresis on SDS-PAGE gels and transferred to PVDF membrane (Bio-Rad Laboratories, Hercules, CA, USA). Membranes were then blocked in 5% fat-free milk for 1 h at room temperature. Thereafter, membranes were incubated with primary antibodies including rabbit anti-MARK4 (4834, dilution 1:1000, Cell Signaling Technology, Danvers, MA, USA), AMPK (5831, dilution 1:1000, Cell Signaling Technology), Phospho-AMPK (2535, dilution 1:1000, Cell Signaling Technology), GAPDH (2118, dilution 1:1000, Cell Signaling Technology), Phospho-NF-κB (3033, dilution 1:1000, Cell Signaling Technology), NF-κB (ab90532, dilution 1:1000, Abcam Biotechnology, Cambridge, MA, USA), Phospho-IKKα/β (2694, dilution 1:1000, Cell Signaling Technology) and ATP5α (ab245580, dilution 1:2000, Abcam Biotechnology) antibody, or mouse anti-IKKα (sc-7606, dilution 1:500, Santa Cruz Biotechnology, Dallas, TX, USA), UQCRC2 (ab14745, dilution 1:2000, Abcam Biotechnology), NDUFB8 (ab110242, dilution 1:1000, Abcam Biotechnology), SDHB (ab14714, dilution 1:2000, Abcam Biotechnology) antibody overnight at 4 °C, followed by incubation with Goat anti-mouse or rabbit IgG horseradish peroxidase (HRP)-conjugated secondary antibodies (HAF007 and HAF008, dilution 1:2000, RD SYSTEMS, Minneapolis, MN, USA) for 1 h at room temperature. Proteins were visualized using the LumiGLO Reagent and Peroxide system (Cell Signaling Technology, Danvers, CO, USA), and then the blots were quantified using Bio-Rad ChemiDoc imaging system (Bio-Rad Laboratories, Hercules, CA, USA). Band density was normalized according to the GAPDH content as previously reported [21].

### 2.9. Statistical Analysis

All the data from cell culture experiments were obtained from at least three independent experiments. Statistical analyses were conducted using SPSS Statistics 20.0 software (IBM SPSS, Armonk, NY, USA). Data were analyzed using One-way ANOVA for comparisons among groups, followed by Duncan test. Results were expressed as means ± SEM. A *p*-value < 0.05 was considered statistically significant, and very significant was indicated when *p* < 0.01.

## 3. Results

### 3.1. MARK4 Promotes Oxidative Stress in Pig Placental Trophoblast Cells

Previous studies in our laboratory and others have shown that an aberrant increase of MARK4 expression is associated with significant placental or adipose oxidative stress in obese model of pig or mice [13,20]. To address whether MARK4 was involved in oxidative stress production of cytotrophoblasts, we first used 400 µM FA to establish the oxidative stress model (Figure 1A–C). As shown in Figure 1D,E, overexpression of MARK4 increased oxidative stress in cytotrophoblasts following 24 h exposure to FA marked by decreased TAC (*p <* 0.05), higher production of ROS (DCF: 52.44% in Myc-MARK4 vs. 34.26% in control, Figure 1E), and elevated protein carbonylation (a marker of protein oxidation) (*p* < 0.05, Figure 1F). These changes were associated with elevated mRNA expression of oxidative stress-related genes, including uncoupling protein 2 (UCP2) and NADPH oxidase 1/4 (NOX1/4) in Myc-MARK4 group, whereas the mRNA content of genes associated with the antioxidant system, including glutathione peroxidase (GPx), catalase (CAT), and superoxide dismutase 2 (SOD2), was reduced in the Myc-MARK4 group compared with vector control group (*p* < 0.05, Figure 1G). In order to further establish the role of MARK4 in modulating oxidative stress of cytotrophoblasts, we next used Vitamin E (2 mM) to alleviate the oxidative stress generation (Figure 1H). As shown in Figure 1I, the addition of VE alleviated the increased ROS production and reduced activities of superoxide dismutase, catalase, and glutathione peroxidase induced by elevated MARK4, while reduced MARK4 allowed the VE to counteract the oxidative stress in pig placental trophoblasts after 24 h FA challenge (*p* < 0.05).

### 3.2. MARK4 Blocks Mitochondrial Oxidative Respiration in Cultured Cytotrophoblasts

**Figure 2:**
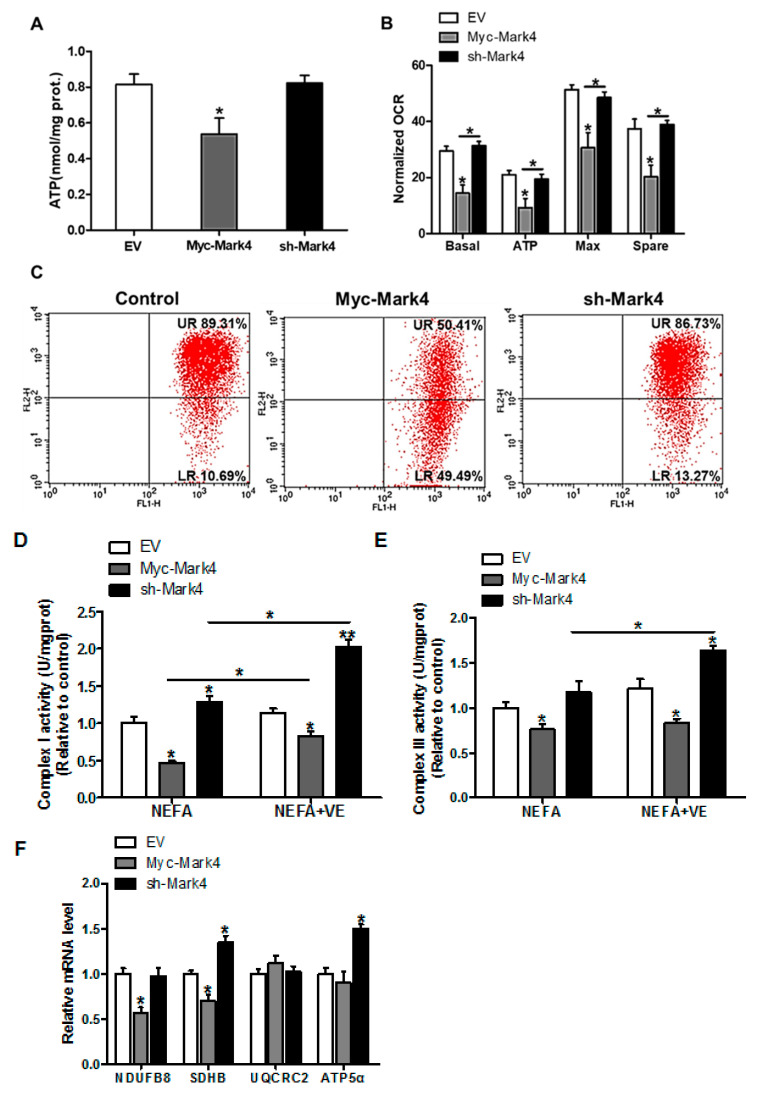
MARK4 blocks mitochondrial oxidative respiration in pig primary trophoblast cells challenged with 400 μM NEFA. (**A**) Mitochondrial ATP production after transfection with Myc-MARK4, sh-MARK4 for 48 h in cytotrophoblasts. Before assessment of ATP generation, cells were incubated with 400 μM NEFA for 24 h (*n* = 3); (**B**) Mitochondrial respiratory parameters were measured in pig cytotrophoblasts after transfection with Myc-MARK4, sh-MARK4 for 48 h. Cells were then treated with 400 μM NEFA for 24 h (*n* = 3). Oxygen consumption rate (OCR) measurements were normalized to total protein level of whole cell lysates extract (pmol O_2_/μg protein); (**C**) Impaired mitochondrial function was estimated by JC-1 fluorescent staining using flow cytometry in cytotrophoblasts (*n* = 3). The upper right (UR) quadrant depicts the percentage of FL-2 (red channel)-positive cells (high mitochondrial membrane potential) and the lower right (LR) quadrant depicts the percentage of FL-2 (red channel)-negative cells (depolarized mitochondrial membrane potential); (**D**,**E**) The activity of mitochondrial complexes I (**D**) and III (**E**) in pig trophoblast cells after transfection with Myc-Mark4, sh-Mark4 for 48 h. Cells were then treated with 400 μM NEFA or 2 mM VE for 24 h (*n* = 3); (**F**) mRNA levels of mitochondrial complexes I (NDUFB8), II (SDHB), III (UQCRC2) and V (ATP5α) (*n* = 3). Values are expressed as mean ± SEM. ** *p* < 0.01; * *p* < 0.05 compared with the control group. NEFA: non-esterified fatty acid; VE: Vitamin E; Myc-MARK4 group: over expression of MARK4 group; sh-MARK4 group: knock down of MARK4 group; Control: empty vector (EV) group.

To gain insight into the mechanisms responsible for the increased oxidative stress induced by elevated MARK4, the mitochondrial oxidative respiration was evaluated in cytotrophoblasts following 24 h exposure to FA. As illustrated in Figure 2A, the ATP production was lower (34% decrease, *p* < 0.05) in the Myc-MARK4 group compared with vector control group or sh-MARK4. In addition to the reduced ATP content induced by overexpression of MARK4, a significant decrease in basal mitochondrial oxygen consumption, ATP-coupled respiration, maximal respiration, and spare capacity were observed in the Myc-MARK4 group compared to the sh-MARK4 or vector control groups (*p* < 0.05, Figure 2B), suggesting that mitochondrial respiration function was compromised by elevated MARK4. Concurrently, Mitochondrial membrane potential determined by JC-1 fluorescent staining, which represented the oxidative respiration level, was decreased in the Myc-Mark4 group (UR: 50.41% in Myc-MARK4 vs. 89.31% in control, Figure 2C). In accordance with reduced mitochondrial oxidative respiration, the activities of mitochondrial Complexes I and III were also decreased in Myc-MARK4 group (*p* < 0.05, Figure 2D,E), and the addition of VE (2 mM) blocked this effect (*p* < 0.05, Figure 2D,E). Moreover, the mRNA expression of genes associated with subunits of mitochondrial complexes I (NDUFB8) and II (SDHB) were significantly reduced in Myc-MARK4 group compared with vector control group (*p* < 0.05, Figure 2F), whereas there was no difference in the mRNA content of mitochondrial complexes III (UQCRC2) and V (ATP5α).

### 3.3. MARK4 Inhibits Mitochondrial Content in Pig Trophoblast Cells

**Figure 3:**
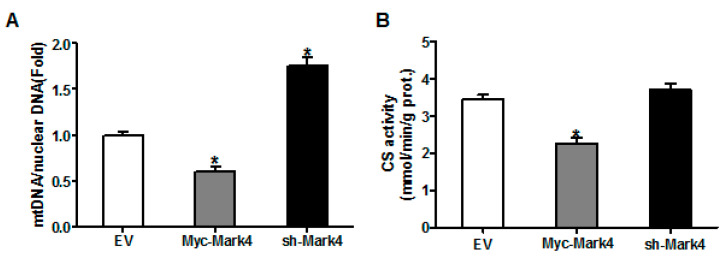

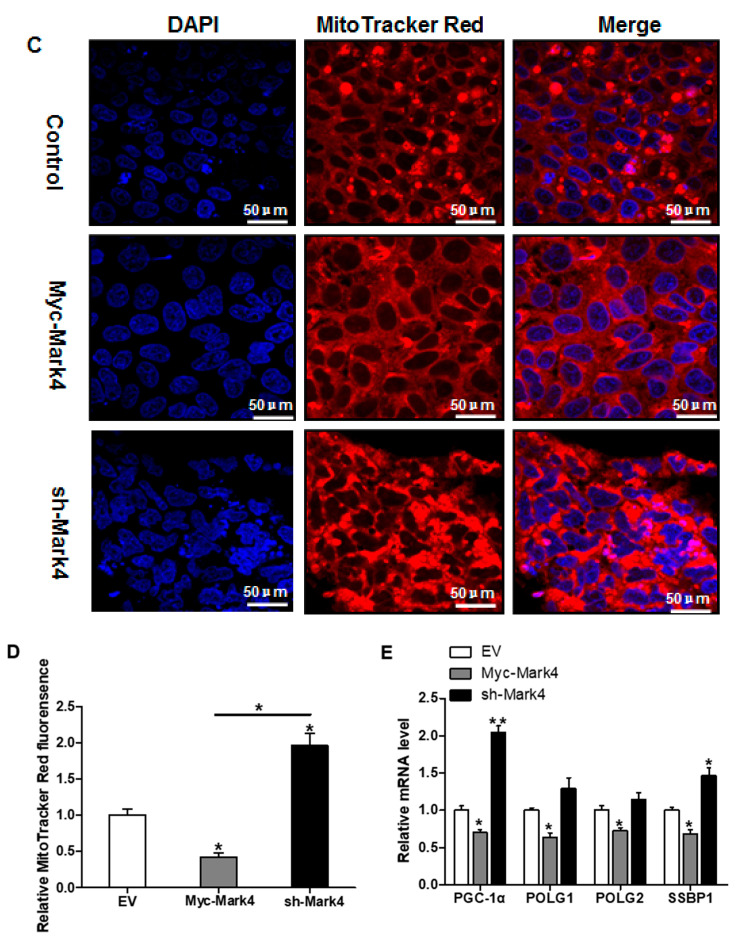
The effect of MARK4 on mitochondrial density in pig primary trophoblast cells challenged with 400 μM NEFA. (**A**,**B**) Mitochondrial biogenesis was estimated by both mtDNA copy number (**A**) and CS activity (**B**) in primary trophoblast cells after transfection with Myc-MARK4 and sh-MARK4 for 48 h. Cells were then incubated with 400 μM NEFA for 24 h (*n* = 3); Representative visualization (**C**) and quantification (**D**) of MitoTracker Red staining for mitochondria after transfection with Myc-MARK4 and sh-MARK4 for 48 h in cytotrophoblasts. Before staining, cells were incubated with 400 μM NEFA for 24 h (*n* = 3). Scale bar: 50 μm; (**E**) mRNA levels of genes implicated in mitochondrial biogenesis and mtDNA replication (*n* = 3). Results were expressed as fold change relative to the values of control cells set to 1 unit. Values are expressed as mean ± SEM. ** *p* < 0.01; * *p* < 0.05 compared with the control group. NEFA: non-esterified fatty acid; mtDNA: mitochondrial DNA; DAPI: 4′, 6-diamidino-2-phenylindole; Myc-MARK4 group: over expression of MARK4 group; sh-MARK4 group: knock down of MARK4 group; Control: empty vector (EV) group.

We next investigated the effects of MARK4 on mitochondrial density in pig placental trophoblast cells. As shown in Figure 3A, the ratio of mitochondrial DNA (mtDNA) to nuclear DNA was significantly decreased in Myc-MARK4 group compared to vector control group, while sh-MARK4 treatment increased mtDNA copy number (*p* < 0.05). In agreement, elevated MARK4 inhibited the citrate synthase (CS) activity (an indicator of mitochondrial content) compared with vector control group (*p* < 0.05, Figure 3B). Concerning mitochondrial biogenesis, we evaluated mitochondrial density assessed by MitoTracker Red fluorescent staining. As illustrated in Figure 3C,D, mitochondrial density was lower in Myc-MARK4 group than in vector control group (*p* < 0.05); this result was also confirmed by transmission electron microscopy, which indicated that the amount of mitochondria in cytotrophoblasts following 36 h exposure to FA was reduced (44% decrease, *p* < 0.05) in Myc-MARK4 group compared with vector control group (Figure S2A,B). Furthermore, the mRNA levels of mitochondria-encoded genes, including, ND1, CYTB, COX1 and ATP6, were significantly decreased in Myc-MARK4 group compared to the control group (*p* < 0.05 or *p* < 0.01, Figure S2C). To gain further insight into the mechanisms by which elevated MARK4 decreased mitochondrial content in pig trophoblast cells challenged with FA, we measured the mRNA levels of genes involved in mitochondrial biogenesis or mtDNA replication and repair. As shown in Figure 3E, the mRNA expression of PGC1α, DNA polymerase subunit 1 (POLG1) and 2 (POLG2) and single-strand DNA binding protein 1 (SSBP1) was lower in Myc-MARK4 group than in vector control group (*p* < 0.05).

### 3.4. Effect of MARK4 on Mitochondrial Ultrastructure in Pig Cytotrophoblasts

**Figure 4:**
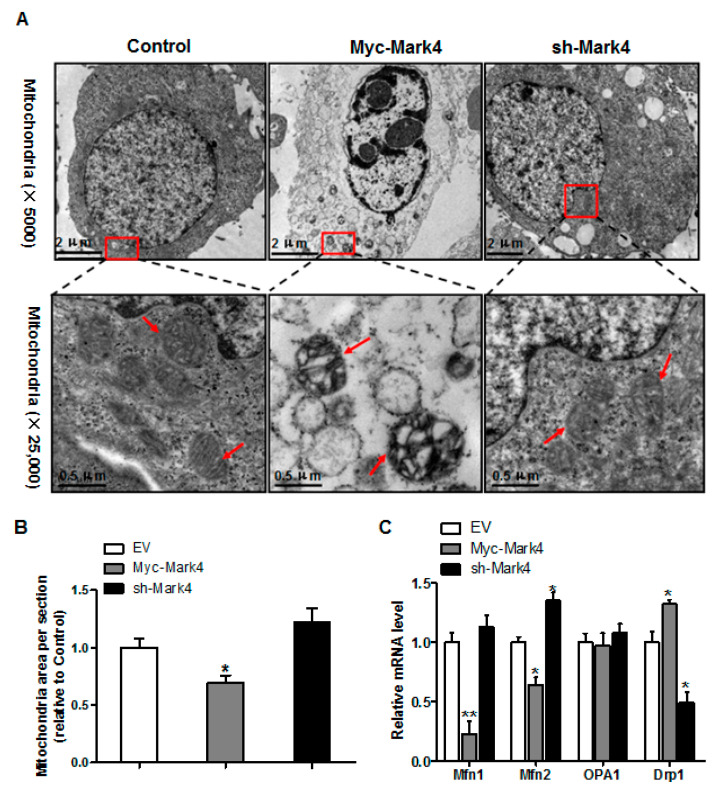
Effects of MARK4 on mitochondrial structure in pig primary trophoblast cells. (**A**) Representative transmission electron microscopy images at original magnifications of ×5000 and ×25,000 in primary trophoblast cells after transfection with Myc-MARK4 and sh-MARK4 for 48 h. Cells were then incubated with 400 μM NEFA for 36 h; (**B**) Quantification of mitochondria area in (**A**) relative to control group (analysis of 10 random images per cell section, *n* = 3); (**C**) Relative mRNA expression of mitochondrial fission and fusion-related regulators after transfection with Myc-MARK4, sh-MARK4 for 48 h in cytotrophoblasts. Cells were then treated with 400 μM NEFA for 36 h (*n* = 3). Values are expressed as mean ± SEM. ** *p* < 0.01; * *p* < 0.05 compared with the control group. NEFA: non-esterified fatty acid; Myc-MARK4 group: over expression of MARK4 group; sh-MARK4 group: knock down of MARK4 group; Control: empty vector (EV) group; Red arrow: mitochondria.

Having determined that mitochondrial density was reduced following Myc-MARK4 + FA treatment, we next addressed whether there was an alteration of mitochondrial structure in response to elevated MARK4. As illustrated in Figure 4A,B, the area of mitochondria was decreased (31% reduction, *p* < 0.05) in Myc-MARK4 group compared with vector control group. Higher magnification (×25,000) further revealed that overexpression of MARK4 negatively altered mitochondrial structure in cytotrophoblasts marked by swollen mitochondria associated with fewer cristae and a decreased electron density of the matrix. Concerning the regulation of mitochondrial dynamic network (maintaining normal mitochondrial morphology), we determined the mRNA levels of genes involved in mitochondrial fission and fusion, including mitofusin 1 (Mfn1), mitofusin 2 (Mfn2), optic atrophy type 1 (OPA1) and Dynamin 1 (Drp1), which contribute to this process [29]. As shown in Figure 4C, elevated MARK4 induced a decrease (*p* < 0.01 or 0.05) in the mRNA content of Mfn1 and Mfn2 (regulating mitochondrial fusion), whereas the mRNA expression of Drp1 (regulating mitochondrial fission) was increased in Myc-MARK4 group compared with the control group (*p* < 0.05). Moreover, the mRNA level of OPA1 (regulating mitochondrial fusion) was not affected by overexpression of MARK4.

### 3.5. MARK4 Blocks Mitochondrial Function and Mitochondrial Biogenesis by Inhibiting the AMPK Pathway in Cultured Cytotrophoblasts

**Figure 5:**
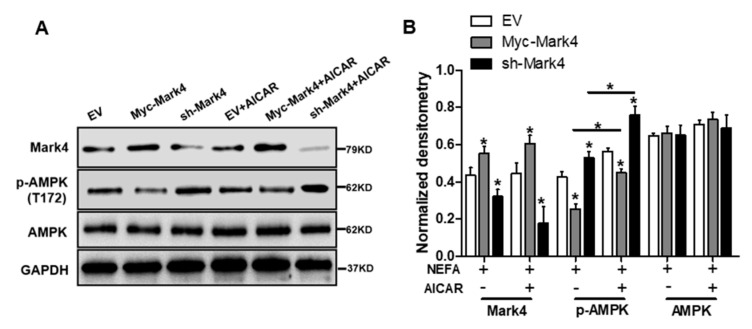

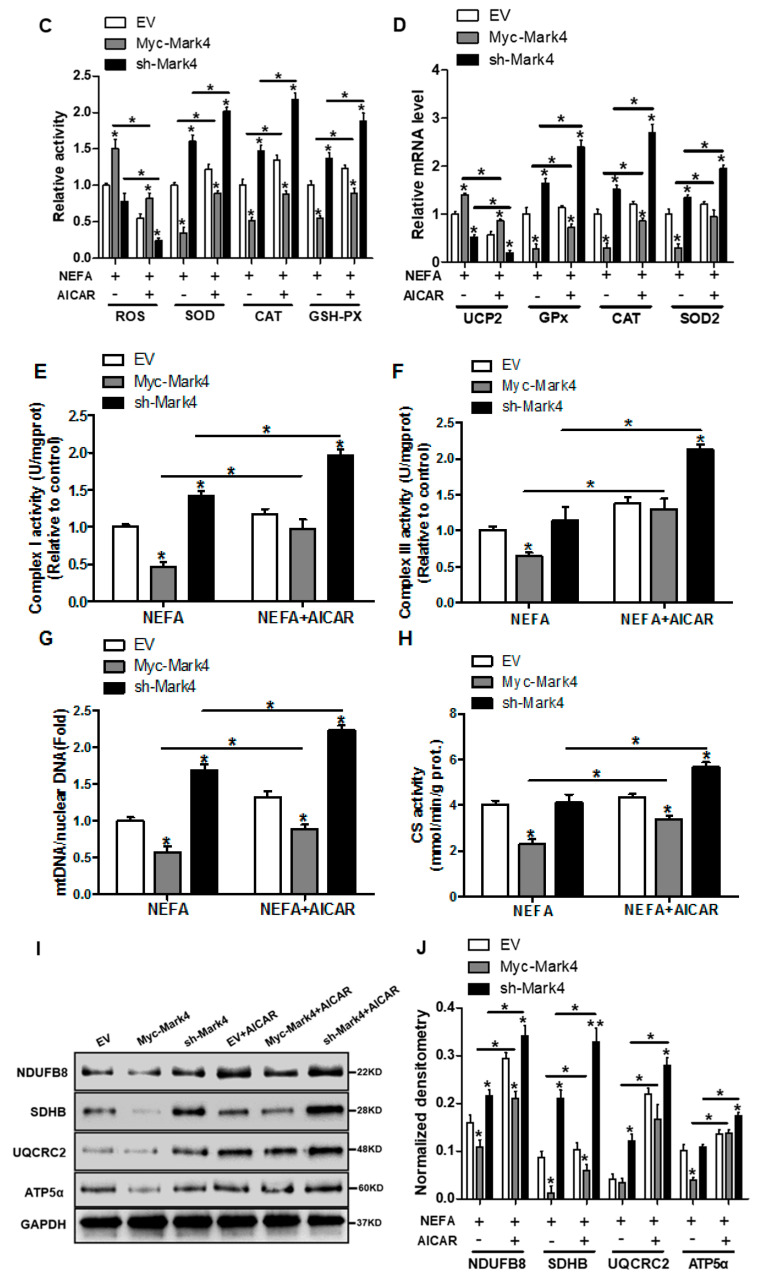

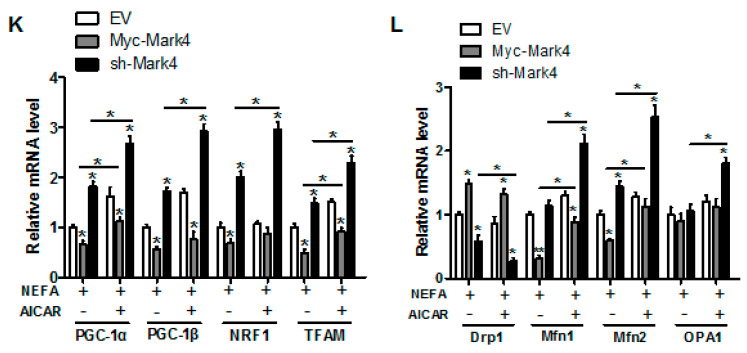
Inhibition of the AMPK pathway by MARK4 blocks mitochondrial function and mitochondrial biogenesis in pig primary trophoblast cells challenged with 400 μM NEFA. (**A**,**B**) Representative immunoblots and densitometric quantification for MARK4, AMPK, and phos-AMPK after transfection with Myc-MARK4 and sh-MARK4 for 48 h in cytotrophoblasts. Cells were then treated with 400 μM NEFA or 1 mM AICAR for 24 h (*n* = 3). (**C**) Relative activity of ROS, SOD, CAT, and GSH-PX after transfection with Myc-MARK4 and sh-MARK4 for 48 h in trophoblast cells. Before the assessment, cells were incubated with 400μM NEFA or 1 mM AICAR for 24 h (*n* = 3). (**D**) mRNA levels of genes implicated in oxidant stress (*n* = 3). Results were expressed as fold change relative to the values of control cells set to 1 unit. (**E**,**F**) The activity of mitochondrial complexes I (**E**) and III (**F**) in pig trophoblast cells (*n* = 3). (**G**,**H**) Mitochondrial biogenesis was estimated by both mtDNA copy number (**G**) and CS activity (**H**) in primary trophoblast cells (*n* = 3). (**I**) Representative immunoblot analysis of mitochondrial complexes I (NDUFB8), II (SDHB), III (UQCRC2), and V (ATP5α). (**J**) Densitometric analysis of corresponding proteins in (**I**) by normalization to GAPDH as an internal control (*n* = 3). (**K**,**L**) Relative mRNA expression of genes implicated in mitochondrial biogenesis (**K**) and mitochondrial fission and fusion (**L**) in cytotrophoblasts. Results are expressed as mean ± SEM. * *p* < 0.05 compared with the control group. ROS: reactive oxygen species; NEFA: non-esterified fatty acid; AICAR: AMPK agonist; Myc-MARK4 group: over expression of MARK4 group; sh-MARK4 group: knock down of MARK4 group; Control: empty vector (EV) group.

It has been shown that obese pregnancy is associated with increased activation of the NF-κB signaling pathway and reduced activity and expression of AMPK in placenta of human beings and animals such as pigs, indicating a potential mechanism for increased placental inflammation and impaired mitochondrial function [1,2,30]. In order to further reveal the molecular mechanism of mitochondrial alterations and dysfunction triggered by MARK4, we first explored whether the AMPK pathway was involved in MARK4-induced oxidative stress and mitochondrial dysfunction in pig trophoblast cells. To verify this hypothesis, we treated cultured cytotrophoblasts with FA in the presence or absence of the AMPK signaling pathway agonist AICAR. As illustrated in Figure 5A,B, western blot analysis revealed that overexpression of MARK4 increased the protein content of MARK4 and inhibited the expression of phos-AMPK (Thr 172) (*p* < 0.05), while no changes were observed in AMPK expression in Myc-MARK4 or sh-MARK4 treatment in the presence or absence of AICAR. Despite with the addition of AICAR, elevated MARK4 still prevented the activation of AMPK (reduced content of phos-AMPK) in cytotrophoblasts after 24 h FA challenge (*p* < 0.05, Figure 5B). Similar to the antioxidant effect of VE, activation of the AMPK signaling pathway by AICAR reduced ROS production in Myc-MARK4 group (DCF: 42.75% in Myc-MARK4 vs. 29.34% in Myc-MARK4 + AICAR, Figure S3A), while activities of glutathione peroxidase, catalase and superoxide dismutase were significantly increased in sh-MARK4 group following AICAR treatment (*p* < 0.05, Figure 5C). Furthermore, higher mRNA content of antioxidant genes, including GPx, CAT and SOD2, was obtained in Myc-MARK4 group following AICAR treatment, along with reduced mRNA level of UCP2 (a marker of increased mitochondrial ROS production) (*p* < 0.05, Figure 5D).

Consistent with decreased oxidative stress in Myc-MARK4 + AICAR treatment, the addition of AICAR promoted the activities of mitochondrial Complexes I and III in the Myc-MARK4 or sh-MARK4 group (*p* < 0.05, Figure 5E,F). Concurrently, Mitochondrial biogenesis indicated by mtDNA copy number, CS activity and mitochondrial density estimated by MitoTracker Red staining was significantly increased in the Myc-MARK4 or sh-MARK4 group following exposure to FA+ AICAR (*p* < 0.05, Figure 5G,H and Figure S3B,C), along with elevated protein contents of NDUFB8, SDHB, UQCRC2, and ATP5α (mitochondrial complexes) and increased mRNA expression of mitochondria-encoded genes, including ATP6, CYTB, ND1, and COX1 (*p* < 0.05, Figure 5I,J and Figure S3D). Moreover, transmission electron microscopy clearly showed that the addition of AICAR for 36 h positively altered a mitochondrial structure (reduced mitochondrial swelling and disruption) in Myc-MARK4 or sh-MARK4 group (Figure S3E). In agreement, the mRNA levels of genes involved in mitochondrial biogenesis, including PGC1α, PGC1β, NRF1, and TFAM, and of genes associated with regulation of mitochondrial morphology, including Mfn1, Mfn2, and OPA1, were higher in the Myc-MARK4 or sh-MARK4 groups after 24 h exposure to FA+ AICAR treatment, along with decreased mRNA content of Drp1 (*p* < 0.05, Figure 5K,L).

### 3.6. IKKα/NF-κB Signal Is Essential for MARK4 Activated Oxidative Stress and Mitochondrial Dysfunction in Pig Trophoblast Cells

**Figure 6:**
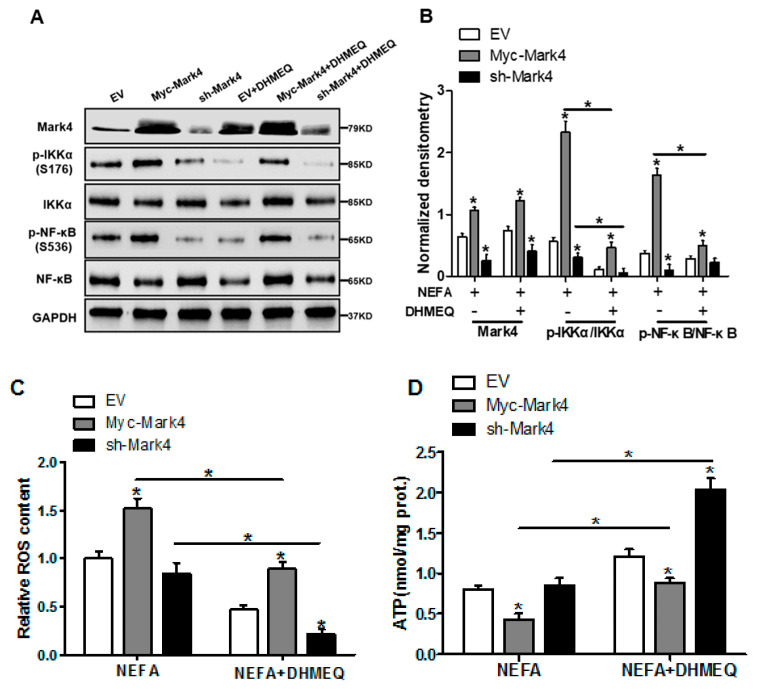

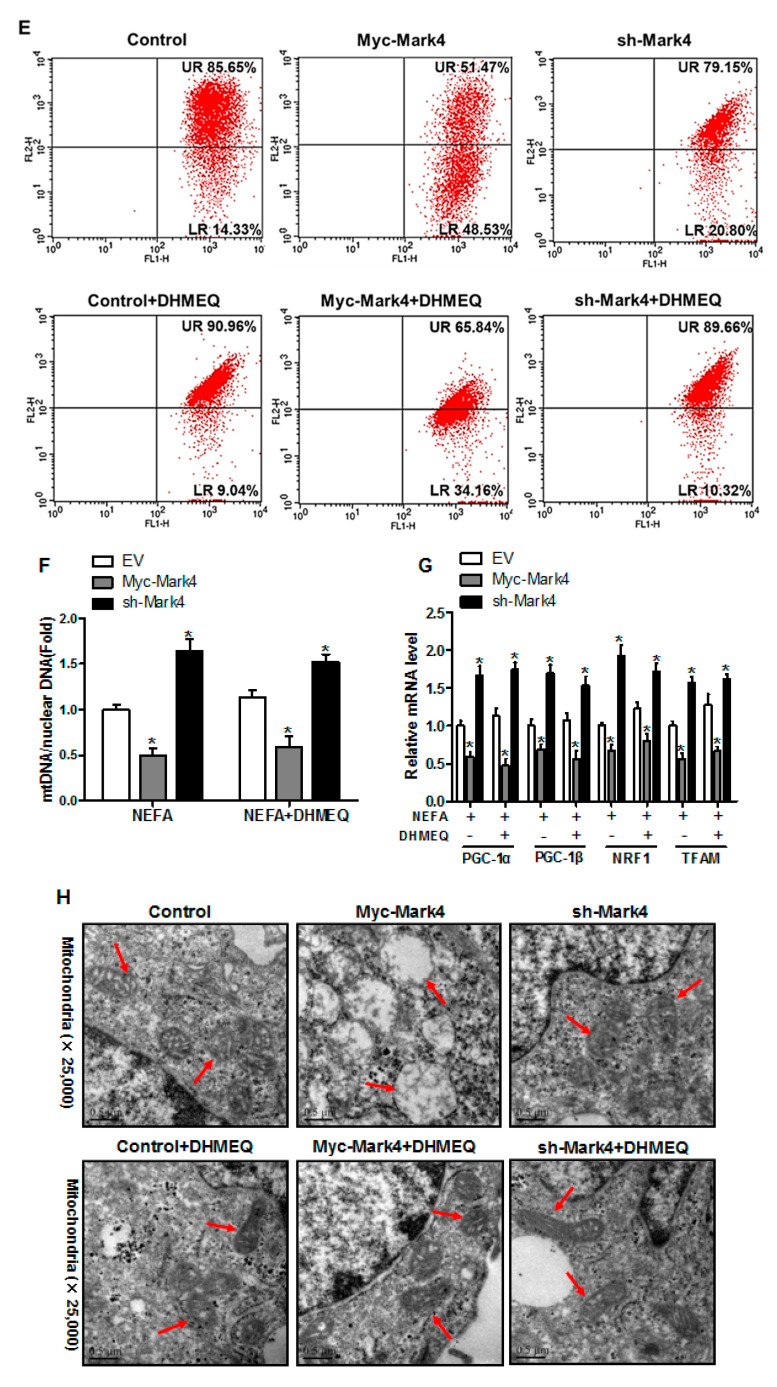

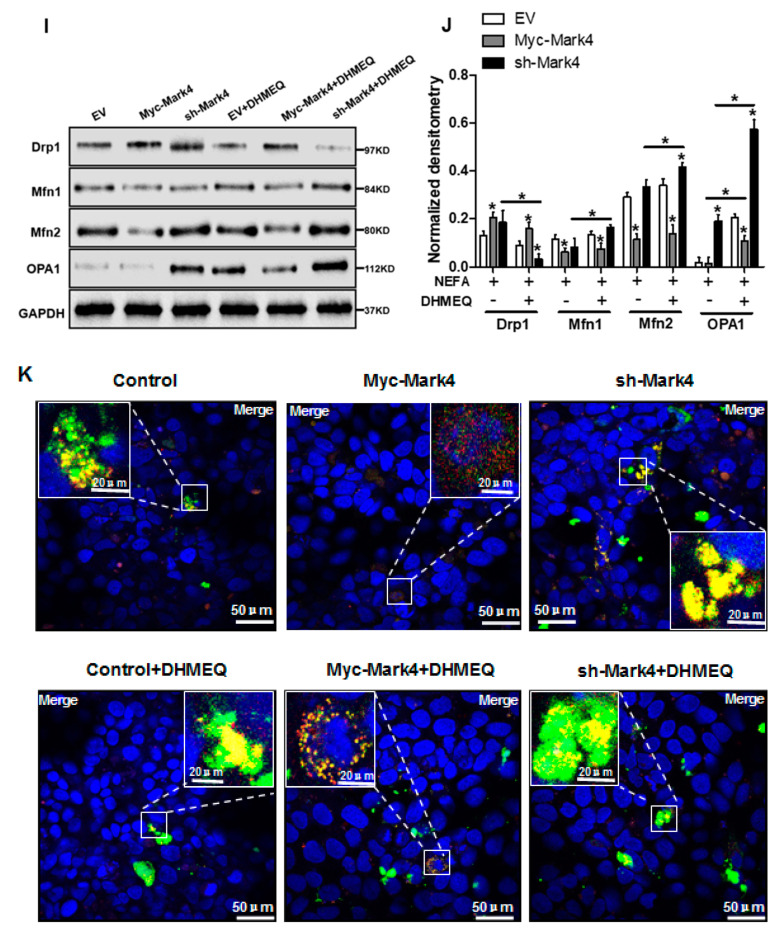
Activation of the NF-κB pathway by MARK4 promotes oxidative stress and mitochondrial dysfunction in pig primary trophoblast cells. (**A**,**B**) Representative immunoblots and densitometric quantification for MARK4, phos-IKKα/IKKα and phos-NF-κB/NF-κB after transfection with Myc-MARK4 and sh-MARK4 for 48 h in cytotrophoblasts. Cells were then treated with 400 μM NEFA or 50 μM DHMEQ for 24 h (*n* = 3). (**C**) ROS production in pig cytotrophoblasts (*n* = 3). (**D**) Mitochondrial ATP production after transfection with Myc-MARK4 and sh-MARK4 for 48 h in cytotrophoblasts. Before the assessment, cells were treated with 400 μM NEFA or 50 μM DHMEQ for 24 h (*n* = 3). (**E**) JC-1 fluorescent staining using flow cytometry in cytotrophoblasts (*n* = 3). (**F**) mtDNA copy number was determined by real-time PCR using the 2^-ΔΔCt^ method in pig trophoblast cells (*n* = 3). (**G**) Relative mRNA expression of genes implicated in mitochondrial biogenesis was determined by quantitative RT-PCR in pig trophoblast cells (*n* = 3). (**H**) Transmission electronic microscopy images (magnification × 25,000) of mitochondria in cytotrophoblasts after transfection with Myc-Mark4 and sh-Mark4 for 48 h. Cells were then incubated with 400 μM NEFA in the presence or absence of 50 μM DHMEQ for 36 h. (**I**) Representative immunoblot analysis of mitochondrial fission and fusion-related regulators. (**J**) Densitometric analysis of corresponding proteins in (**I**) by normalization to GAPDH as an internal control (*n* = 3). (**K**) The assessment of mitochondrial function associated with fission and fusion in cytotrophoblasts after transfection with Myc-MARK4, sh-MARK4, mito-pEGFP, and mito-DsRed2 for 48 h. Cells were then incubated with 400 μM NEFA or 50 μM DHMEQ for 24 h. Scale bar: 50 μm or 20 μm. Values are expressed as mean ± SEM. * *p* < 0.05 compared with the control group. ROS: reactive oxygen species; NEFA: non-esterified fatty acid; DHMEQ: NF-κB specific-inhibitor; Myc-MARK4 group: over expression of MARK4 group; sh-MARK4 group: knock down of MARK4 group; Control: empty vector (EV) group. Red arrow: mitochondria.

We next explored whether the IKKα/NF-κB pathway was involved in MARK4 activated oxidative stress and mitochondrial dysfunction in pig cytotrophoblasts. Specifically, overexpression of MARK4 elevated the ratio of phosphorylated NF-κB (Ser536) to total NF-κB, accompanying increased IKKα phosphorylation (Ser176) (*p* < 0.05, Figure 6A,B). Despite with DHMEQ treatment which is a specific NF-κB pathway inhibitor, elevated MARK4 prevented DHMEQ from alleviating activation of IKKα/NF-κB signal (increased content of phos-IKKα/NF-κB) in pig trophoblast cells following 24 h exposure to FA (*p* < 0.05, Figure 6B). In addition, suppression of NF-κB by DHMEQ treatment reduced ROS production in Myc-MARK4 or sh-MARK4 group (*p* < 0.05, Figure 6C). Consistently, ATP content was increased in Myc-MARK4 or sh-MARK4 group following FA+ DHMEQ treatment (*p* < 0.05, Figure 6D), as well as mitochondrial membrane potential assessed by JC-1 fluorescent staining was elevated in DHMEQ treatment (UR: 51.47% in Myc-MARK4 vs. 65.84% in Myc-MARK4 + DHMEQ, Figure 6E). Moreover, the addition of DHMEQ did not alter mtDNA copy number in Myc-MARK4 or sh-MARK4 group (Figure 6F), similar to the mRNA content of genes associated with mitochondrial biogenesis, including PGC1α, PGC1β, NRF1, and TFAM (Figure 6G). Conversely, abnormal mitochondrial morphology was recovered (reduced mitochondrial swelling and disarrayed cristae) in Myc-MARK4 group after exposure to FA+ DHMEQ treatment (Figure 6H). Protein expression of Mfn1, Mfn2, and OPA1 were also up-regulated in the Myc-MARK4 or sh-MARK4 groups following DHMEQ treatment, accompanying reduced protein content of Drp1 (*p* < 0.05, Figure 6I,J).

We further confirmed the role of IKKα/NF-κB signaling in MARK4 activated mitochondrial dysfunction in pig placental trophoblast cells by mitochondrial fusion analysis. To investigate mitochondrial fusion in this trial, we transfected cytotrophoblasts in each group with DNA constructs expressing green (mtEGFP) or red (mtDsRed2) fluorescent proteins targeted to the mitochondrial matrix, respectively. Considering the mitochondrial presequence of matrix proteins is cleaved during import reaction, we then conducted cell fusion (cells in each group with differently labeled mitochondria) with PEG400 in the presence of cycloheximide to inhibit newly synthesized mtEGFP/mtDsRed2 (see Materials and Methods). Therefore, these experiments estimated the mitochondrial function associated with fission and fusion by exchanging fluorescent matrix proteins between mitochondria, instead of the cytosol through successive export and import reactions. As illustrated in Figure 6K, four hours after cell fusion, the fraction of double-labeled mitochondria (yellow) was increased in Myc-MARK4 group following FA+ DHMEQ treatment, demonstrating the exchange of fluorescent matrix proteins by mitochondrial fusion. Collectively, our data indicated the IKKα/NF-κB pathway participated in MARK4 activated oxidative stress and mitochondrial dysfunction in pig placental trophoblast cells.

## 4. Discussion

Ectopic lipid accumulation (lipotoxicity) induced by hyperlipidemia has been shown to induce oxidative stress, mitochondrial dysfunction and inflammation in highly metabolic tissues like liver and muscle in various obese models [5,31]. Recently, the hypothesis that Lipotoxicity directly contributes to metabolic dysfunction in placental tissues has gained increased attention. MARK4, a member of the AMP-activated protein kinase (AMPK)-related family of kinases, has been reported to positively regulate adipogenesis and apoptosis in mice adipocytes, as well as adipose oxidative stress and inflammation [19,20]. In addition, our previous studies showed that maternal obesity (excessive back-fat) induces lipotoxicity in the full-term pig placenta [2], along with increased activation of MARK4 [21], and our in vitro cell experiment further revealed that MARK4 promotes lipogenesis by activating WNT/β-catenin and inhibiting PPARγ pathways in pig placental trophoblasts, suggesting that MARK4 may be involved in metabolic dysfunction associated with excessive back-fat in the pig placenta [23]. Regarding the impact of MARK4 on metabolic dysfunction, we initially hypothesized that MARK4 may potentially stimulate oxidative stress in other cell types besides porcine placental trophoblast cells, and this study was designed to investigate the role of MARK4 in modulating the redox status in porcine placental trophoblasts in vitro. We found that Mark4 significantly increased production of ROS and protein carbonylation and reduced SOD, CAT, and GSH-PX activities in pig trophoblast cells, suggesting elevated oxidative stress by MARK4 overexpression. Thus, our data indicated that MARK4 is involved in regulating mitochondrial oxidative stress in pig placental trophoblasts upon the status of lipotoxic insult.

There is currently cumulative evidence showing that adverse alterations in mitochondrial respiration and enzymatic activity of mitochondrial complexes in the human placenta are involved in pregnancy loss complicated by maternal obesity [9,12]. Indeed, similar to highly metabolically active tissues like adipose and muscle, the placenta is also susceptible to obesity-associated oxidative stress, resulting in mitochondrial damage associated with placental dysfunction and therefore the poor fetal development [7,10]. Furthermore, our previous data indicated that excessive back-fat of sows during pregnancy contributes to placental mitochondrial defects induced by exaggerated oxidative stress, along with increased activation of MARK4 in pig placenta, suggesting that MARK4 may play an important role in regulating ROS-mediated mitochondrial abnormalities in pig placenta associated with excessive back-fat [13]. Thus, in this trial, we next focused on the influence of MARK4 on mitochondrial oxidative injury. By treating an in vitro model of pig trophoblast cells with fatty acid, we induced mitochondrial dysfunction, as expected MARK4 aggravated this process by reducing ATP generation, decreasing mitochondrial membrane potential, and lowering mitochondrial Complexes I and III activities. Although this effect was blocked by VE, an antioxidant, MARK4 still significantly impaired mitochondrial oxidative respiration and induced oxidative stress. In addition, several studies have suggested that as well as mitochondrial function, mitochondrial biogenesis is also altered in the human or pig term placenta associated with maternal obesity [12,21]. Consistent with our expectation, our results revealed that activation of oxidative stress caused by MARK4 led to decreased mitochondrial number (reduced mtDNA content and CS activity) associated with a concomitant reduction in expression of genes related to mtDNA replication (POLG1, POLG2, and SSBP1) in pig cytotrophoblasts. Furthermore, Studies reported that abnormalities of mitochondrial structure may contribute to decreased mitochondrial content through disruption of mitochondrial dynamic network [32]. Consistent with this notion, we found that elevated MARK4 caused structural abnormalities of the mitochondria (mitochondrial swelling and membrane disruption) in cultured pig trophoblast cells, as revealed by electron microscopy. It is noted that maintaining normal mitochondrial structure depends on mitochondrial biogenesis and continuous cycles of fission and fusion [33]. Accordingly, we observed that enhanced MARK4 contributed to decreased mRNA content of mitochondrial fusion regulator (Mfn1 and Mfn2) associated with increased expression of Drp1 (participating in the mitochondrial fission), which suggested that aggravated mitochondrial fission caused by MARK4 overexpression may contribute to reduced mitochondrial content in pig placental trophoblasts, potentially by disruption of mitochondrial structures. From these findings, we surmise that MARK4 may elicit a causal mediator of mitochondrial injury in pig placenta induced by excessive back-fat.

As a member of the LKB1 family of kinases, AMPK has been most intensively studied due to its key role in regulating energy homeostasis and metabolic disease [34]. AMPK has been demonstrated to promote muscle glucose uptake and fatty acid oxidation and to reduce hepatic glucose production and lipogenesis [35]. Recently, several studies reported that activation of AMPK prevents lipid induced lipotoxicity in retinal pericytes [36]. In agreement, our previous data showed that excessive back-fat is associated with reduced activity and expression of AMPK in pig term placenta, indicating a potential mechanism for increased placental lipid accretion and oxidative stress [2]. Indeed, AMPK is also found to be involved in regulating mitochondrial biogenesis and function by AMP-activated protein kinase-induced activation of PGC-1α [37]. Our finding showed that MARK4 inhibited the activation of AMPK and the mRNA expression of transcription factors associated with mitochondrial biogenesis, including PGC-1α, PGC-1β, NRF1, and TFAM, and protein content of mitochondrial complexes in cultured pig trophoblast cells, suggesting compromised mitochondrial biogenesis and function, as evidenced by significantly decreased mitochondrial density determined by MitoTracker Red fluorescent staining and impaired mitochondrial oxidative respiration for pig trophoblasts in vitro by elevated Mark4. Recent studies indicated that AMPK and MARK4 play an opposing role in adipose or muscle glucose and energy homeostasis [18]. Consistently, we preliminarily demonstrated that activation of AMPK by AMPK agonist AICAR prevented MARK4 from stimulating oxidative stress and mitochondrial abnormalities in respiration function, content, and structure in cultured pig trophoblast cells in response to lipotoxic insult, which suggests that MARK4 may promote oxidative stress and mitochondrial dysfunction in pig trophoblasts by inhibiting the AMPK signaling pathway.

There exists a substantial amount of evidence supporting that obese pregnancy is associated with increased circulating fatty acids and inflammatory cytokines and an exaggerated pro-inflammatory state in human or pig placenta, which may impair mitochondrial function through activation of the NF-κB signaling pathway [2,30,38]. The NF-κB signal is a cytosolic sensor which can be activated by IKK induced phosphorylation of regulatory protein IκB. Then NF-κB molecules translocate to nucleus and promote target genes transcription [39]. As previously documented, the IKKα/NF-κB signal pathway is involved in MARK4 regulation of oxidative stress and inflammation in mice adipocytes, suggesting that Mark4 could be a positive regulator of the NF-κB pathway in mammalian cells [20]. In support of a significant correlation between MARK4 and NF-κB signaling, our data showed that elevated MARK4 increased the phosphorylation of IKKα/NF-κB signal pathway, while DHMEQ, a specific inhibitor of NF-κB signaling, attenuated this effect. Although there was not a significant difference in mtDNA content, increased oxidative stress and mitochondrial injury induced by MARK4 overexpression was blocked by the addition of DHMEQ, in agreement with the previous studies that pro-inflammatory cytokine TNF-α induces mitochondrial abnormalities in 3T3-L1 adipocytes without obvious effects on mitochondrial biogenesis [40]. In this trial, inhibition of NF-κB signaling by DHMEQ also recovered abnormal mitochondrial morphology (decreased mitochondrial hollow and increased cristae) induced by MARK4 in cultured trophoblast cells. Mitochondrial morphology is closely correlated to mitochondrial function and metabolic activity [41]. Therefore, activation of the NF-κB pathway by MARK4 could be a possible mechanism involved in placental mitochondrial injury associated with increased back-fat. In addition, previous studies have shown that mitochondrial metabolism and function is supported by the dynamics of this organelle, which is controlled by mitochondrial fusion and fission [42]. Accordingly, we demonstrated that the addition of DHMEQ alleviated heightened mitochondrial fission induced by MARK4 in pig trophoblast cells in vitro, suggesting that the NF-κB signal pathway is central to MARK4 activating dynamic morphology changes observed and therefore mitochondrial dysfunction in pig trophoblasts in response to lipotoxic insult. However, the precise mechanism for activation of NF-κB signal in regulating mitochondrial dynamics perturbation of pig trophoblasts induced by MARK4 needs to be further studied.

## 5. Conclusions

**Figure 7:**
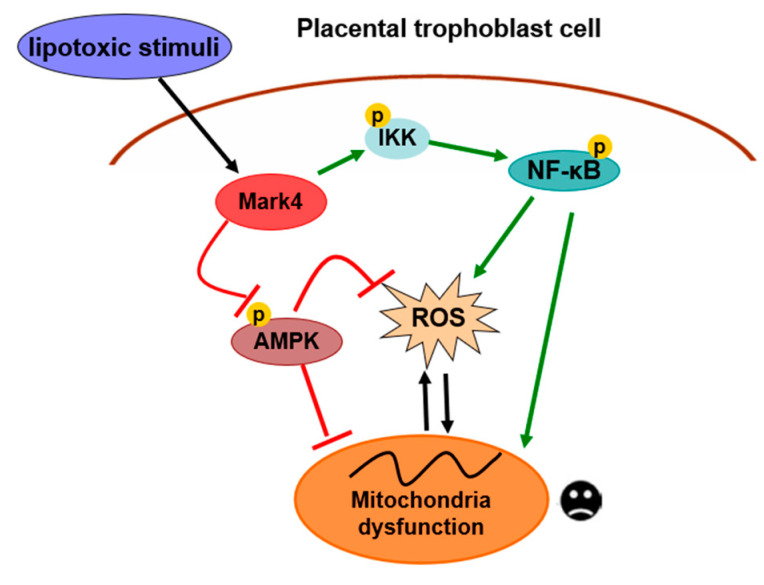
A proposed model for role of MARK4 in the regulation of oxidative stress and mitochondrial dysfunction in pig placental trophoblast cells. MARK4 promotes oxidative stress and mitochondrial dysfunction via activating IKKα/NF-κB and inhibiting AMPK signaling pathways. Arrows indicates a positive regulation and bar-headed lines show negative regulation. Interactions depicted are based on studies performed in various tissues (in some cases placenta) and have been previously published. ROS: reactive oxygen species; P: phosphorylation.

In summary, our present study demonstrates that MARK4 stimulates oxidative stress and incites mitochondrial abnormalities in porcine trophoblast cells upon the status of lipotoxic insult, which could contribute to placental dysfunction in conditions associated with excessive back-fat during pregnancy of sows. Moreover, both AMPK and NF-κB signals are involved in MARK4 activated oxidative stress and mitochondrial dysfunction in pig placental trophoblasts (Figure 7). Thus, our results indicate that MARK4 has potential as a regulator of mitochondrial injury associated with excessive back-fat in the pig placenta, and anti-MARK4 therapy targeted to inhibit oxidative stress and mitochondrial injury in cytotrophoblasts may serve as a novel therapeutic strategy for treatment of obesity-associated pregnant complications.

## Supplementary Materials

The following are available online at https://www.mdpi.com/article/10.3390/biomedicines10010165/s1. Figure S1: The selection of sh-MARK4 vector and MARK4 transfection efficiency detection. Figure S2: MARK4 decreases mitochondrial content in pig primary trophoblast cells challenged with 400 μM NEFA. Figure S3: AMPK-mediated mitochondrial biogenesis is blocked by MARK4 in pig primary trophoblast cells. Table S1: Primer sets used for real-time PCR.
