# Supplementary material for: Microtubule Affinity-Regulating Kinase 4 Promotes Oxidative Stress and Mitochondrial Dysfunction by Activating NF-κB and Inhibiting AMPK Pathways in Porcine Placental Trophoblasts
Source: Biomedicines. 2022 Jan 13;10(1):165. doi: 10.3390/biomedicines10010165 (PMC8773735; doi:10.3390/biomedicines10010165)
Supplement: Supplementary file 1 [file biomedicines-10-00165-s001.zip › Figure S1.pdf]

Figure S1

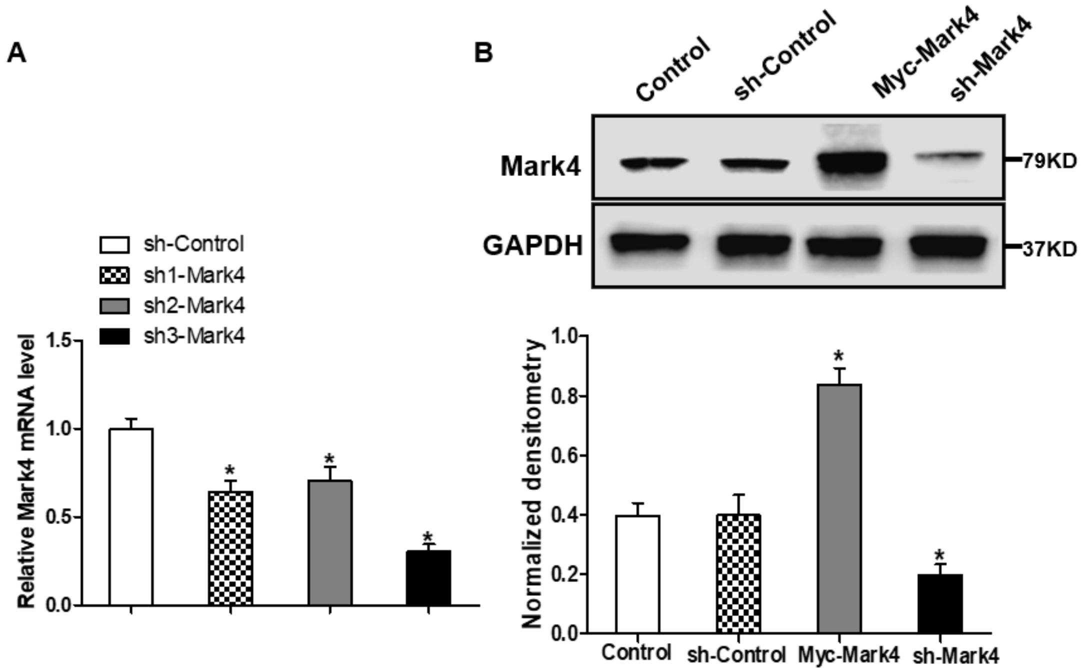

Figure S1. The selection of sh-MARK4 vector and MARK4 transfection efficiency detection. (a) Relative mRNA expression level of MARK4 after transfection with sh-Control, sh1- MARK4, sh2- MARK4 and sh3- MARK4 for 24h in cytotrophoblasts (n = 3). To exclude off-target effects of shRNA treatment, three shRNAs of MARK4 was constructed and synthesized by Genepharma Company (Shanghai, China) using pGPU6/Neo shRNA expression vector; (b) Representative immunoblots and densitometric quantification for MARK4 after transfection with Myc-MARK4, sh-MARK4 and sh-Control for 48h in cytotrophoblasts (n = 3). The level of total GAPDH was determined as loading control. Results are expressed as mean ± SEM. * $p < 0.05$ compared with the control group. Myc-MARK4 group: over expression of MARK4 group; sh-MARK4 group: knock down of MARK4 group; Control: empty vector (EV) group; sh-Control: transfected with negative-shRNA group.
