# Supplementary material for: Microtubule Affinity-Regulating Kinase 4 Promotes Oxidative Stress and Mitochondrial Dysfunction by Activating NF-κB and Inhibiting AMPK Pathways in Porcine Placental Trophoblasts
Source: Biomedicines. 2022 Jan 13;10(1):165. doi: 10.3390/biomedicines10010165 (PMC8773735; doi:10.3390/biomedicines10010165)
Supplement: Supplementary file 1 [file biomedicines-10-00165-s001.zip › Figure S2.pdf]

Figure S2

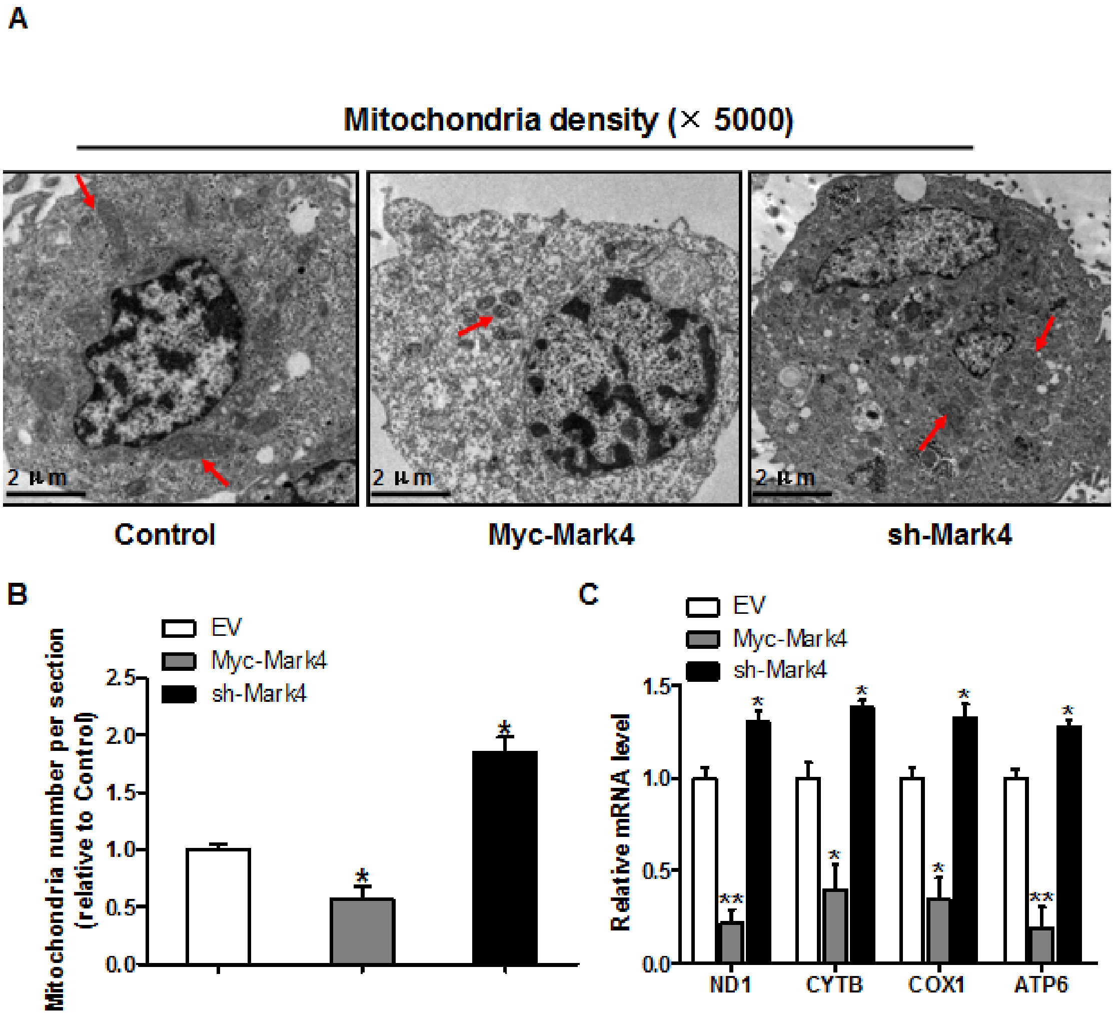

Figure S2. MARK4 decreases mitochondrial content in pig primary trophoblast cells challenged with 400 μM NEFA. (a) Mitochondrial density assessed by electron microscopy in cytotrophoblasts after transfection with Myc-MARK4 and sh-MARK4 for 48h. Cells were then incubated with 400μM NEFA for 36h. Original magnification, ×5,000; (b) Quantification of mitochondrial number per image area in (a) relative to control group (analysis of 10 random images per cell section, n = 3); (c) The relative mRNA level of various mitochondria-encoded genes was determined by quantitative RT-PCR in pig trophoblast cells (n = 3). Results were expressed as fold change relative to the values of control cells set to 1 unit. Values are expressed as mean ± SEM. ** $P < 0.01$; * $P < 0.05$ compared with the control group. NEFA: non-esterified fatty acid; Myc-MARK4 group: over expression of MARK4 group; sh-MARK4 group: knock down of MARK4 group; Control: empty vector (EV) group; Red arrow: mitochondria.
