# Supplementary material for: Microtubule Affinity-Regulating Kinase 4 Promotes Oxidative Stress and Mitochondrial Dysfunction by Activating NF-κB and Inhibiting AMPK Pathways in Porcine Placental Trophoblasts
Source: Biomedicines. 2022 Jan 13;10(1):165. doi: 10.3390/biomedicines10010165 (PMC8773735; doi:10.3390/biomedicines10010165)
Supplement: Supplementary file 1 [file biomedicines-10-00165-s001.zip › Figure S3.pdf]

# Figure S3

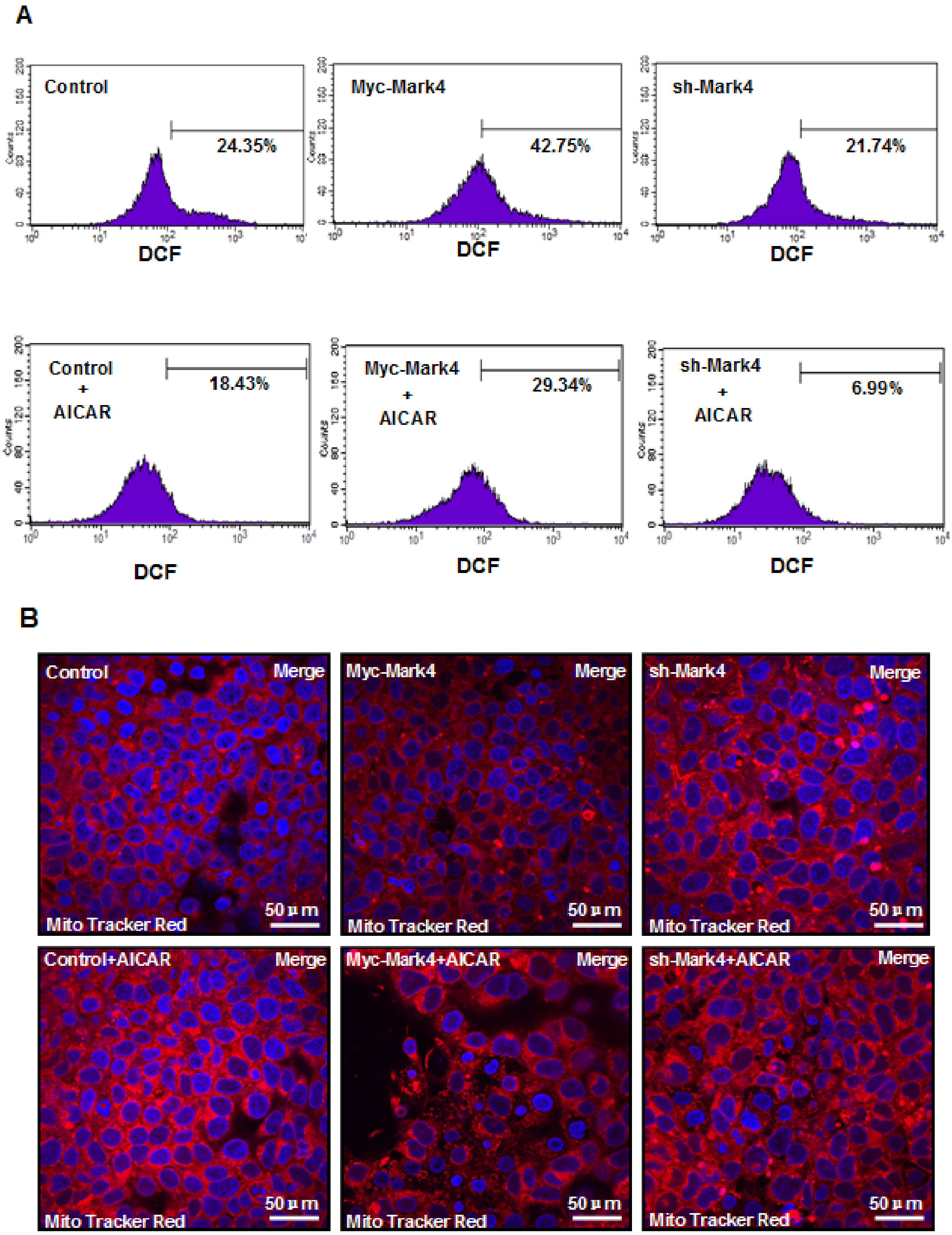

A
Control
24.35%
Myc-Mark4
42.75%
sh-Mark4
21.74%
Control
+
AICAR
18.43%
Myc-Mark4
+
AICAR
29.34%
sh-Mark4
+
AICAR
6.99%
DCF
Counts
B
Control
Myc-Mark4
sh-Mark4
Control+AICAR
Myc-Mark4+AICAR
sh-Mark4+AICAR
Merge
Mito Tracker Red
50 μm

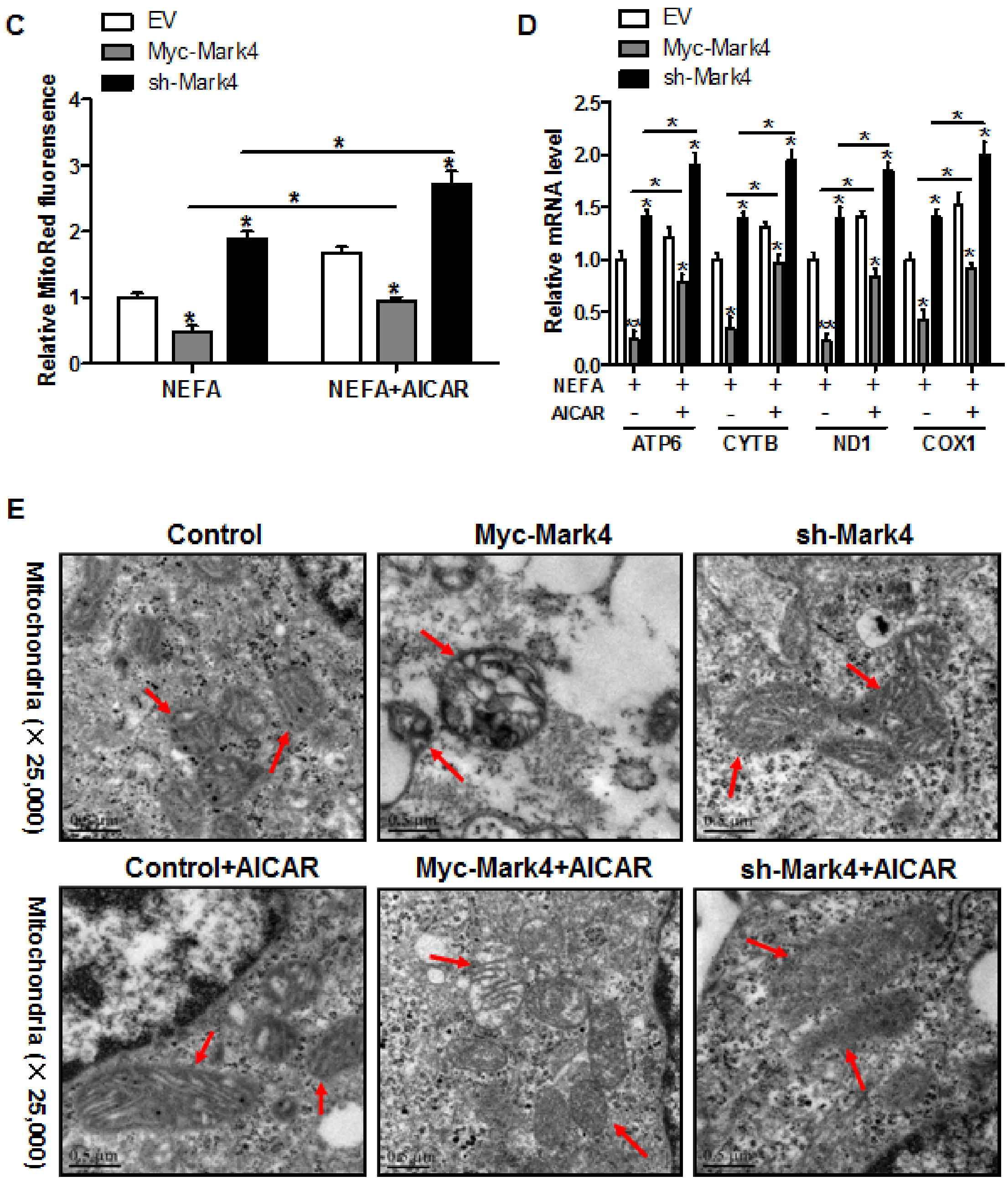


**Figure S3.** AMPK-mediated mitochondrial biogenesis is blocked by MARK4 in pig primary trophoblast cells. (a) ROS generation was measured by DCF production using flow cytometry in pig cytotrophoblasts after transfection with Myc-MARK4 and sh-MARK4 for 48h. Before the assessment, cells were incubated with 400μM NEFA or 1 mM AICAR for 24h (n = 3). Value in scale bar depicts the percentage of DCF-positive cells; (b) Mitochondrial biogenesis was estimated by MitoTracker Red staining for mitochondria after transfection with Myc-MARK4 and sh-MARK4 for 48h in cytotrophoblasts. Before staining, cells were incubated with 400μM NEFA or 1 mM AICAR for 24h. Scale bar: 50μm; (c) Quantification of MitoTracker Red staining in (b) by measuring the fluorensence intensity relative to control group (analysis of 10 random microscopic fields per cell section, n = 3); (d) mRNA levels of various mitochondria-encoded genes in cytotrophoblasts (n = 3); (e) Transmission electronic microscopy images (magnification × 25,000) of mitochondria in cytotrophoblasts after transfection with Myc-MARK4, sh-MARK4 for 48h. Cells were then incubated with 400 μM NEFA in the presence or

absence of 1 mM AICAR for 36 h. Values are expressed as mean ± SEM. ** $p < 0.01$; * $p < 0.05$ compared with the control group. NEFA: non-esterified fatty acid; AICAR: AMPK agonist; DCF: dichlorofluorescein; Myc-MARK4 group: over expression of MARK4 group; sh-MARK4 group: knock down of MARK4 group; Control: empty vector (EV) group. Red arrow: mitochondria.
