# Supplementary material for: Microtubule Affinity-Regulating Kinase 4 Promotes Oxidative Stress and Mitochondrial Dysfunction by Activating NF-κB and Inhibiting AMPK Pathways in Porcine Placental Trophoblasts
Source: Biomedicines. 2022 Jan 13;10(1):165. doi: 10.3390/biomedicines10010165 (PMC8773735; doi:10.3390/biomedicines10010165)
Supplement: Supplementary file 1 [file biomedicines-10-00165-s001.zip › Table S1.pdf]

Table S1 Primer sets used for real-time PCR

Gene name†	Productsize (bp)	Direction	Sequence, 5′-/- 3′
UCP2	119	Forward:	CACCCAATGTCGCTCGTAATG
		Reverse:	CGTGAAGTGGCAGGGAAGGT
GPx	149	Forward:	ATGAGCTTTAGCCGCCTGTT
		Reverse:	TCGATGTCCTTGGCTGAGAA
CAT	127	Forward:	AACAGTGCCAACGAAGATAA
		Reverse:	GGATGAACAGTTGTGCGTCT
SOD2	129	Forward:	TCTGGACAAATCTGAGCCCTAA
		Reverse:	CTTGGACGCCGACGGATACA
ATP6	131	Forward:	ATCACTAGCCCACTTTCTAC
		Reverse:	CTGCTGTAATGTTGGCTGTC
ND1	94	Forward:	GGCACATCCTCAATCTCCAT
		Reverse:	AGAGGGTAGGGTATTGGTAG
Cyclophilin-A	127	Forward:	AGGATTTATGTGCCAGGGTG
		Reverse:	CATGGACAAGATGCCAGGAC
PGC-1α	126	Forward:	CAACAATGAGCCTTCAAACA
		Reverse:	GCATCAAATGAGGGCAATCC
TFAM	122	Forward:	AAGCTCAGAACCCAGATGCA
		Reverse:	CACCTGCCAGTCTGCCCTAT
NRF1	82	Forward:	GTCCTTTGTATCTCACCCTCC
		Reverse:	ACTTACGCACCACATTCTCC
MFN1	132	Forward:	CGATGGGCATCATTGTTGTT
		Reverse:	TCTCCTTGGCACGGGTGGTC
COX3	85	Forward:	CTCCTCCAAGCCTCAGAATA
		Reverse:	ACCCTGTAGCCACAAAGAAA
CYTB	86	Forward:	ATCGGAACAGACCTCGTAGA
		Reverse:	ATAAAGTGGAAGGCGAAGAA
PGC-1β	113	Forward:	TGCCAAAGAAGCACCCAGAGC
		Reverse:	GTCACCAAACGAGCAGGAGAAGG
Drp1	100	Forward:	TAAACCGAAGCCAGAAGGACA
		Reverse:	AAGTGGCGATAGGAAGGGTG
MFN2	128	Forward:	CTGTGGGCTGGAGACTCATCG
		Reverse:	GCTGGCGTACTCTACAAACTGG
OPA1	117	Forward:	ATGAAATAGAACTCCGAATG
		Reverse:	GTAAGTCAACAAGCACCATC
COX1	100	Forward:	GGAGCGGGTACTGGATGAAC
		Reverse:	CTGCAAGGTGTAGGGAGAAA
Mark4	138	Forward:	TCCGAGAAGTCCGCATCA
		Reverse:	GCGACACGAGGTAGTCAAACAC
NDUFB8	134	Forward:	GGCCTTCATGCTGTTCAT
		Reverse:	CCACTGGCTCAGGTTCTTT
SDHB	125	Forward:	TGTGGTCCTATGGTGTTGG
		Reverse:	TTGCCTCCGTTGATGTTC

Gene name†	Productsize (bp)	Direction	Sequence, 5′-/- 3′
UQCRC2	135	Forward:	ATTCTGGACTCTTCGGGATT
		Reverse:	TGTTCTTGGCAGCTTGGA
ATP5α	110	Forward:	TGCCACTCAACAACTCTT
		Reverse:	CACCCGCATAGATAACAG
NOX1	136	Forward:	CCCTTTACCCTGACCTCT
		Reverse:	GACCATCCACCTCAATCC
NOX4	99	Forward:	TCCGAATGGTCAAAGAAA
		Reverse:	TGAGGGTAAATGGATGGT
POLG1	118	Forward:	AGCATCCATGACGAGGTTCGC
		Reverse:	TCAGACCCAGCTTGTAGGCAAA
POLG2	90	Forward:	CTTCAGCAGCAGTGATTGTC
		Reverse:	GTTTCTATTGGCTCCTTTCC
SSBP1	124	Forward:	ATACCAAATGGGCGATGTCA
		Reverse:	TTTCCCTTCCACGTAAACTC
GAPDH	233	Forward:	CCACAACATACGTAGCACCACGATC
		Reverse:	CCTTCATTGACCTCCACTACATGGT
HPRT1	131	Forward:	TCAAGCAGCATAATCCAAAG
		Reverse:	AAGGGCATAGCCTACCACAA

†All porcine gene sequences.
